# Pesticide Encapsulation at the Nanoscale Drives Changes to the Hydrophobic Partitioning and Toxicity of an Active Ingredient

**DOI:** 10.3390/nano9010081

**Published:** 2019-01-09

**Authors:** Matthew Slattery, Bryan Harper, Stacey Harper

**Affiliations:** 1 Department of Environmental and Molecular Toxicology, Oregon State University, Corvallis, OR 97330, USA; slatterm@oregonstate.edu; 2 School of Chemical, Biological and Environmental Engineering, Oregon State University, Corvallis, OR 97330, USA; bryan.harper@oregonstate.edu; 3 Oregon Nanoscience and Microtechnologies Institute, Corvallis, OR 97330, USA

**Keywords:** nanopesticide, pyrethroid, nanoenabled, other ingredients, pesticide risk assessment, nanotoxicology, encapsulated

## Abstract

Given the costs associated with designing novel active ingredients, new formulations focus on the use of other ingredients to modify existing formulations. Nanosized encapsulated pesticides offer a variety of enhanced features including controlled release and improved efficacy. Despite the presence of nanosized capsules in current-use pesticide formulations, the analytical and toxicological implications of encapsulation are uncertain. To explore this issue quantitatively, we fractionated the capsules of a commercially available encapsulated insecticide formulation (γ-cyhalothrin active ingredient) into two size ranges: a large fraction (LF), with an average hydrodynamic diameter (HDD) of 758 nm, and a small fraction (SF), with an average HDD of 449 nm. We developed a novel extraction method demonstrating a time-dependent inhibition of γ-cyhalothrin from capsules for up to 48 h. An acute immobilization test with a freshwater macroinvertebrate (*Ceriodaphnia dubia*) revealed that the SF was significantly more toxic than both the LF and the free γ-cyhalothrin treatment (EC50 = 0.18 µg/L, 0.57 µg/L, and 0.65 µg/L, respectively). These findings highlight that encapsulation of γ-cyhalothrin mitigates hydrophobic partitioning in a time-dependent manner and influences toxicity in a size-dependent manner. Recognizing the analytical and toxicological nuances of various nanosized capsules can contribute to innovation in pesticide formulations and may lead to more comprehensive pesticide regulation.

## 1. Introduction

More than 55 billion dollars are spent every year on pesticides [1], reflecting their enormous economic and agricultural importance. While pesticide products are typically composed of many chemicals, in the United States, they are regulated according to their active ingredients—the primary drivers of their targeted toxicity. Development and registration of a novel active ingredient is costly and requires up to two years of review [2]. This incentivizes manufactures to implement other ingredients (also known as inert ingredients) when reformulating pesticides rather than create new active ingredients. Other ingredients in pesticide products face less regulatory scrutiny, sparking contention over their capacity to modify a pesticide’s risk to nontarget organisms [3,4,5,6]. Nonetheless, reformulation of existing active ingredients is a common practice in the pesticide industry, as indicated by the multitude of distinct products containing identical active ingredients.

Among the innovative formulations available on the market, encapsulated pesticides offer a variety of desirable features that include reduction in human exposure to active ingredients, controlled release, longer residual concentrations, elimination of organic solvents, and increased efficacy [7,8,9]. Encapsulation technologies utilize a three-dimensional barrier that surrounds active ingredients, shielding them from immediate interaction with their surrounding chemical environment. However, these capsules may also result in incompatibilities with current pesticide risk assessments. For example, chemical descriptors for hydrophobicity (K_OW_, the octanol–water partitioning coefficient) and soil sorption (K_d_, the soil adsorption coefficient) may not be reflective of an active ingredient once encapsulated [10]. Pesticide fate and transport models are highly dependent on these descriptive parameters of active ingredients, and can suffer from significant uncertainty when these values are inaccurate [11,12].

Further complicating our understanding of encapsulation technologies, capsules in the nanoscale (1–1000 nm) are garnering attention in pesticide literature [13,14,15,16] and have been observed in current-use formulations [17,18]. Nanoencapsulated formulas capitalize on the sheer diversity of nanomaterials available, including polymers, lipids, mesoporous silica, clay, and other materials [19,20,21]. In addition to many capsule compositions, the size of nanoscale capsules is likely a relevant factor in pesticide formulations, given that nanoparticle size is typically a driving factor in their colloidal behavior and increased relative surface area [22]. While nanospecific effects are recognized in pesticide formulations [23], little is known about the influence of capsule size at the nanoscale.

As nanoencapsulation technologies infiltrate the pesticide market, it is important to note that nanoenabled products can display anomalous behavior when compared to their conventional counterparts [23] and present unique incompatibilities with pesticide regulation [24]. This has drawn concern over integration with current legislative framework [25]. Pyrethroid-based active ingredients appear to be particularly responsive to behavior modifications resulting from nanoenabled formulations [23]. The effects associated with both nanoenabled and encapsulated pesticides could exacerbate discrepancies between expected and observed concentrations, especially when combined as a nanoencapsulated formulation. A quantitative method to assess the extent of capsule-based interference on hydrophobic partitioning would help address concerns that encapsulation renders conventional descriptors such as K_OW_ misleading. Additionally, supplementing this data with toxicity data is necessary to address the demand for comparisons between nan-enabled pesticide formulations and their conventional counterparts [23].

This study sought to investigate these issues by (i) isolating and separating nanosized capsules from a current-use, pyrethroid-based insecticide using centrifugation and passive settling; (ii) designing a novel, time-dependent extraction technique intended for laboratory extraction and hydrophobic comparison of active ingredients and their encapsulated counterparts; and (iii) conducting an acute toxicity test to compare the response of a freshwater macroinvertebrate, *Ceriodaphnia dubia*, to freely suspended and nanoencapsulated active ingredients.

## 2. Materials and Methods

### 2.1. Chemicals

A commercially available, EPA-registered capsule suspension insecticide (EPA Reg. No. 67760-104-53883) with 5.9% γ-cyhalothrin was used. Analytical standard grade γ-cyhalothrin [3-(2-chloro-3,3,3-triuoro-1-propenyl)-2,2-dimethyl,cyano(3-phenoxyphenyl) methyl ester], 98.5% purity (CAS number 68085-85-8) was purchased from Crescent Chemical Company (Islandia, NY, USA). Hexane (CAS number 110-54-3) was purchased from Avantor Performance Materials, Inc. (Center Valley, PA, USA).

### 2.2. Preparation of Capsule Fractions from Commercial Product

The encapsulated pesticide formulation used here is sold as a concentrated product (59.9 g γ-cyhalothrin/L according to the label) which was diluted with ultrapure Milli-Q water (Milli-Q Gradient A10 water purification system equipped with a Q-Gard^®^ 2 and a Quantum™ IX Ultrapure Organex cartridge, Millipore Corp., Billerica, MA, USA) to a 100 mg/L stock. In order to isolate the pesticide capsules in the formula and reduce the presence of other ingredients, 10 mL aliquots of the stock were centrifuged for 30 min at 7000 g with a benchtop Eppendorf 5430 centrifuge (Hamburg, Germany), creating a capsule pellet. Carefully, 9 mL of the resulting supernatant was removed from each aliquot and replaced with Milli-Q water, then vortexed to resuspend the capsule pellet to form the unfractionated (UF) samples. To separate the UF capsules into two distinct size ranges, the UF samples were centrifuged again for 7 mins at 1400 g. This produced a supernatant containing the small fraction (SF), while the large fraction (LF) was generated by resuspending the capsule pellet in Milli-Q water. To ensure that the capsules remained in suspension and did not form aggregates over time, the SF and LF samples were set undisturbed in the dark for 48 h before carefully collecting the top 5 mL of the settled SF and LF samples, which were stored in a glass vial at 4 °C for later analysis.

### 2.3. Capsule Characterization

The hydrodynamic diameter (HDD), zeta potential, and polydispersity index (PDI) of the three fractions (UF, SF, and LF) were measured in triplicate by dynamic light scattering (DLS) using a Zetasizer Nano ZS (Malvern Instruments, LTD., Worcestershire, UK) at 25 °C. Statistical differences between fractions were determined with a one-way ANOVA. Analyses were considered significantly different at *p* ≤ 0.05. The size and morphology of capsules were verified using an FEI Quanta 600 FEG (FEI CO., Hillsboro, OR, USA) scanning electron microscope (SEM) operating at 10 kV using samples prepared by dropping 20 µL of each fraction onto a Si substrate and drying before imaging.

### 2.4. Quantification and Partitioning of γ-cyhalothrin

An Agilent Technologies 7820A GC (Agilent Technologies, Santa Clara, CA, USA) equipped with an Agilent Technologies 7963A Automatic Liquid Sampler, electron capture detector (ECD), and 25 m × 0.32 mm ID BPX35 column was used to quantify γ-cyhalothrin in test samples. Standard grade γ-cyhalothrin was run at 0.05, 0.1, 1.0, and 2.5 mg/L to produce a calibration curve and verify retention times at the beginning of every run. Solutions of LF and SF were diluted with Moderately Hard Water (MHW) to approximately 1 mg/L for γ-cyhalothrin quantification. MHW was prepared according to the Environmental Protection Agency (EPA) recipe [26]. The LF and SF solutions were compared against a free γ-cyhalothrin (FC) treatment, prepared by adding γ-cyhalothrin suspended in acetone to MHW such that the concentration of γ-cyhalothrin was 1 mg/L and the concentration of acetone was 1 mL/L. Then, 600 µL of hexane was mixed with 600 µL of LF, SF, or FC in 1.5 mL gas chromatography (GC) vials in triplicate. Vials were placed on a SCILOGEX MX-T6-S Analog Tube Roller set to 30 rpm for 2 minutes (0 h), 1, 6, 24, 48, or 72 h. After agitation, 500 µL of hexane was aspirated from the vials and diluted with 500 µL of fresh hexane in a new GC vial for analysis. Preliminary studies demonstrated complete recovery of γ-cyhalothrin from the full formulation (quantification of γ-cyhalothrin by GC-ECD that matched concentrations listed on the product label) at 72 h, and therefore γ-cyhalothrin recovery is presented as a percentage of the 72 h concentration. A two-way ANOVA analysis was used to compare γ-cyhalothrin recovery among capsule type and extraction time. Analyses were considered significantly different at *p* ≤ 0.05.

### 2.5. Daphnia Immobilization Assay

*Ceriodaphnia dubia* less than 24 h old were provided by the Aquatic Toxicology Laboratory at Oregon State University, cultured in MHW according to EPA specifications [26]. Toxicity assessments at 48 h used immobility of *C. dubia* as an endpoint, defined as no observable swimming action for 15 s under gentle agitation of the test vessel (although the heart may still be beating or antennae moving). A total of five treatments were tested: MHW control, 1 mL/L acetone control (in MHW water), FC, SF, and LF. The γ-cyhalothrin concentrations in the FC, SF, and LF stock solutions were verified by GC-ECD before dilution with MHW to concentrations of 0.006, 0.017, 0.05, 0.45, 1.35, 4.05, and 12.5 µg/L. For each treatment and control, 10 *C. dubia* were placed individually in glass vials with 2 mL of their respective solution. There were two experimental replicates per concentration and control, for a total of 20 neonates per treatment. The test period was 48 h under 18 h light period with full-spectrum lights and the water temperature remained at 20 °C.

A one-way ANOVA was used to compare differences between experimental replicates. We used the *drc* package in R, version 3.0-1, to generate a two-parameter log-logistic model of the *C. dubia* dose–response curve that was compared pairwise using a one-way ANOVA. Estimates of the EC50 (concentration required for 50% immobilization) for the FC, LF, and SF treatments were calculated using *drc*. These estimates were compared using a one-way ANOVA test. Analyses were considered significantly different at *p* ≤ 0.05.

## 3. Results and Discussion

### 3.1. Capsule Isolation, Fractionation, and Characterization

**Figure 1:**
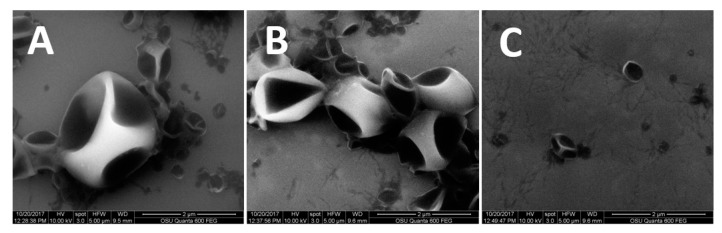
Representative SEM images of the encapsulated pesticide in each of the formulation fractions, showing the capsule morphology and size ranges. Scale bars are 2 µm. UF (**A**), LF (**B**), and SF (**C**).

The SEM images of the UF, LF, and SF show a collapsed, spherical capsule morphology in all fractions (Figure 1). Presumably, the capsules collapse once dried and placed in the vacuum of the SEM chamber. In aqueous suspension, this may be a cavity where the capsule payload is located. According to the pesticide’s marketing materials, the capsules are designed to dispense γ-cyhalothrin in two stages [27]. There is an initial rapid release from the disruption of thin-walled capsules, followed by a slow release through thick-walled capsules that preserve the active ingredient for multiple weeks. Variability in wall thickness could not be verified by the SEM, though the shape and structure of the capsules is aligned with this description.

We compared the diameter of the capsules, indicated by the average hydrodynamic diameter (HDD); their electrophoretic mobility, indicated by the zeta potential; and their heterogeneity, indicated by the polydispersity index (PDI). The HDD of all fractions was less than 1000 nm, and each fraction was significantly different from the others, ranked in increasing order as SF < UF < LF (Table 1). This demonstrates that the fractionation method employed here successfully separated the UF into two distinct nanoscale capsule fractions. While previous studies have investigated differences between micro- and nanocapsules [17], this study benefits from comparisons within the nanoscale to better understand the relative importance of capsule size when formulated below 1000 nm.

When comparing heterogeneity, the PDIs of all three treatments were below 0.3 (Table 1), indicating a suitably monodisperse sample for DLS measurement. The UF was significantly different from the LF, which would be expected if the fractionation process was successfully separating the UF into increasingly homogenous nanoscale capsule fractions. This was corroborated by the SEM images, in which the UF (Figure 1A) shows the greatest diversity of capsule sizes. Zeta potential was not different for any of the capsule treatments (Table 1).

It is noteworthy to mention that the 48 h settling period was necessary to allow particularly large capsules (>1000 nm) to settle out of suspension. This is important for accurate DLS measurements, which can be disrupted by the presence of settling particles in a sample. The settling process also ensured that the LF and SF capsules were stable in suspension throughout the duration of a 48 h static aquatic exposure. We determined that the LF and SF together constitute approximately 2% of the total γ-cyhalothrin contained in the full pesticide formulation. This suggests that the nanosized capsules are not a majority constituent of the original product although 2% could be an underestimate due to the potential loss of capsules during the fractionation process.

The isolation and fractionation method employed here is simple and scalable, and allowed us to reliably separate capsules according to their size. Processes like this can be used to investigate other encapsulated formulas, facilitating further research into the effects of capsule size with different capsule compositions or active ingredients. Further, separation processes during manufacturing could be leveraged by the pesticide industry to develop tightly controlled formulations with specific sizes for novel applications.

### 3.2. γ-Cyhalothrin Partitioning

**Figure 2:**
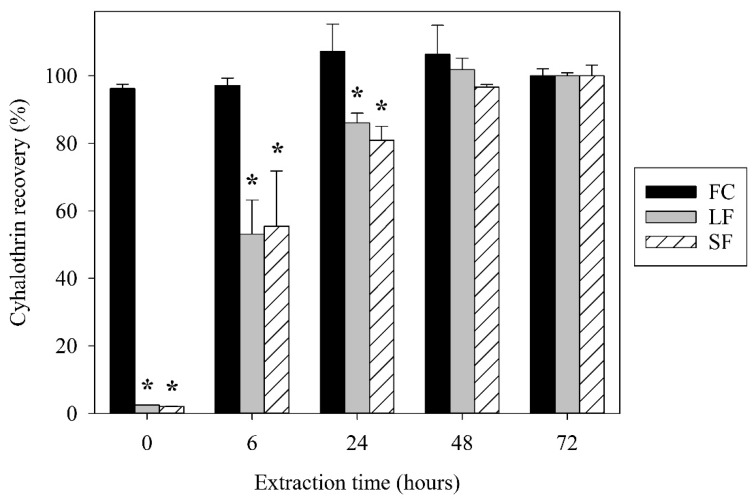
Recovery of total γ-cyhalothrin from FC, LF, and SF treatments at different extraction times. Bars represent standard error. * indicates significant difference from FC (*p* ≤ 0.05).

Recovery of free γ-cyhalothrin, determined by GC-ECD quantification of γ-cyhalothrin after the extraction process, was immediate and consistent from 0 to 72 h (Figure 2), indicating uninhibited hydrophobic partitioning of free γ-cyhalothrin out of the aqueous sample and into hexane. This is unsurprising considering that pyrethroids are deliberately designed to be hydrophobic to minimize their transport in water [28]; thus, γ-cyhalothrin’s extremely low solubility in water (<3 µg/L) and high solubility in hexane (>500 g/L) is expected to drive strongly hydrophobic behavior.

In contrast, γ-cyhalothrin recovery from the LF and SF was less than 3% at 0 h (Figure 2). The recovery of these encapsulated treatments increased over time, plateauing around 48 h. There was a significant difference between both encapsulated treatments and the FC at 0, 6, and 24 h. This suggests that initially, only a small fraction of the total γ-cyhalothrin in the LF and SF is freely available for partitioning into the nonpolar phase. This agrees with the notion of an internal capsule cavity that contains a bulk of the active ingredient, and the percentage of surface bound and freely dissolved γ-cyhalothrin is less than 3% at 0 h. As time progresses from 0 to 48 h, the capsules degrade until all the γ-cyhalothrin is released. Preliminary trials showed consistently low recoveries of γ-cyhalothrin at 0 h for SF and LF samples that were multiple days old, suggesting that the capsules are relatively stable in aqueous solution without the presence of a nonpolar extraction solvent. Interestingly, there were no significant differences in γ-cyhalothrin recovery between the LF and SF at any point, suggesting that these capsules interfere with hydrophobic partitioning similarly regardless of their size.

Interpreting these findings with respect to pesticide regulation highlights potential inconsistencies with regulatory strategies focused on the behavior of active ingredients. For example, implementing K_OW_ in pesticide transport modeling is meant to account for the hydrophobic partitioning of the active ingredient. However, we have shown that encapsulation can inhibit the hydrophobic behavior of a pyrethroid for up to 48 h when in the presence of a nonpolar phase. This time dependency may be an important consideration when modeling pesticide transport in the days following an application, when the hydrophobicity of an active ingredient is still shielded by the capsules. For example, γ-cyhalothrin may be more mobile during a rainfall event for the first two days when formulated in nanosized capsules. While the capsules are assumed to release the relatively immobile γ-cyhalothrin into the desired area over time, a risk assessment may not address the movement of the capsules themselves prior to the release of γ-cyhalothrin. Determining the fate and transport of the capsules themselves is complicated by both the proprietary nature of capsule composition and the challenges associated with distinguishing engineered nanoparticles from complex environmental media.

The time-dependent extraction method utilized here allowed us to make quantitative comparisons between encapsulated products and their freely suspended counterparts. It uses a noncarcinogenic solvent, minimal sample preparation, and low sample volumes. Developing methodology like this is a necessary step towards understanding how the fate and behavior of pesticides can be altered by nanosized encapsulated formulations. Building on these methods and screening additional capsule designs will address the current shortage of data regarding nanoenabled pesticides [25]. It is possible that existing shortcomings in nanopesticide regulation affect our ability to mitigate environmental exposures of hydrophobic active ingredients, especially in light of the widespread contamination of pyrethroids in surface waters that exceed regulatory thresholds and predictions [29]. Such discrepancies between modeled and measured environmental pyrethroid concentrations could indicate that current risk assessments are inadequate for accurate predictions about active ingredients. One approach to resolving this issue may be to incorporate an initial kinetics step that accounts for the release of an active ingredient from its nanoparticle carrier. Notably, this step will be susceptible to factors beyond the typical concentration and rate coefficients delineated by the active ingredient, given that the physiochemical parameters of the pesticide are not informative of the capsule diameter or thickness.

### 3.3. Size-Dependent Immobilization of C. dubia

In our 48 h immobilization test with *C. dubia*, the response from control treatments was 10% or less. The lack of response from the acetone control affirmed that acetone was a suitable carrier solvent for γ-cyhalothrin in our FC samples, allowing for exposures above the pyrethroid’s solubility limit. There was no significant difference between experimental replicates, so response data was aggregated.

**Figure 3:**
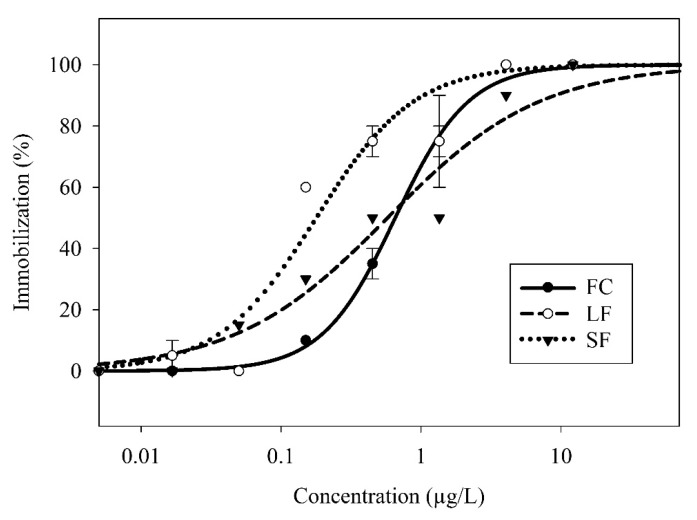
Two-parameter log-logistic regressions of *C. dubia* immobilization response to FC, LF, and SF as a function of γ-cyhalothrin concentration. Symbols represent sample means, bars represent standard error.

**Figure 4:**
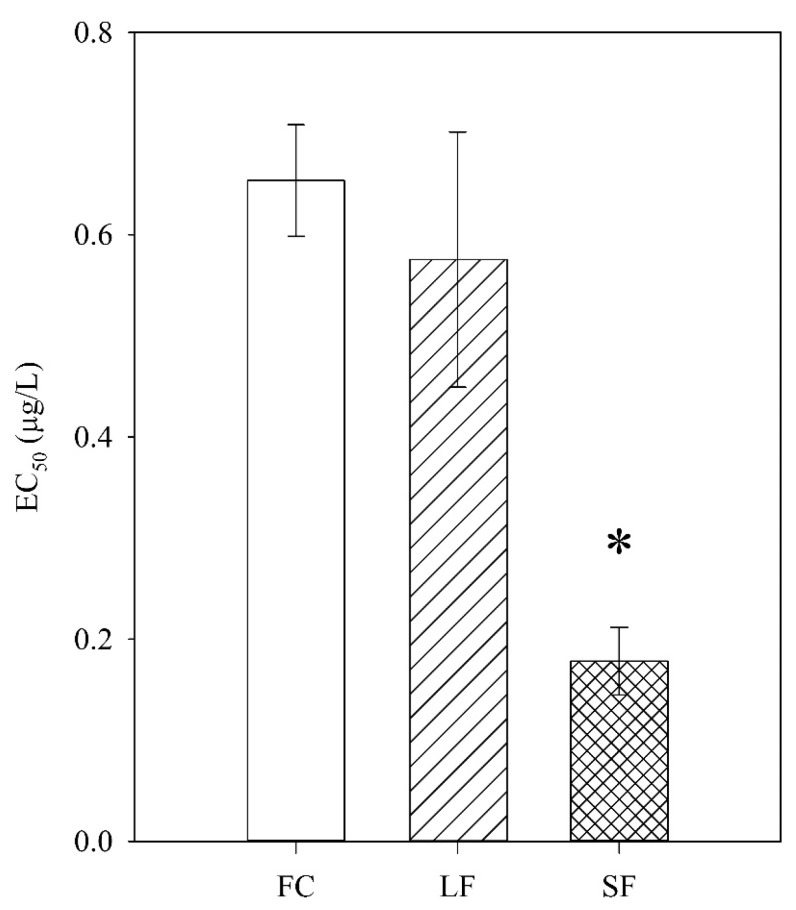
Estimated EC50 values for FC, LF, and SF. Bars represent standard error. * indicates significant difference from FC (*p* ≤ 0.05).

The *C. dubia* showed a dose-dependent response to the FC, SF, and LF exposures as modeled by a two-parameter, log-logistic regression (Figure 3). The SF showed the highest toxicity (EC50 = 0.18 µg/L), followed by the LF (EC50 = 0.57 µg/L) and the FC (EC50 = 0.65 µg/L) (Figure 4). The SF, but not the LF, was significantly more toxic than the FC. This indicated a size-dependent effect on toxicity. Considering the proprietary nature of the capsule composition, it is impossible to eliminate a synergistic or additive effect due to the capsules themselves—a capsule-only control is impossible without cooperation with the manufacturer. However, if the LF and SF capsules have identical chemical makeup, the insignificant difference between the LF and the FC makes synergistic effects unlikely. If the capsules themselves indeed elicited a toxic response, there are many biocompatible capsule materials available for use as nanocarriers [30] that might eliminate capsule toxicity.

When accounting for slope, all three modeled curves were significantly different from each other. The slope of the response curves increases in order of LF < SF < FC. The increased response variability observed in the LF and SF samples, as indicated by their lower slope values, could be explained by changes in the distribution of γ-cyhalothrin throughout the exposure solution. In the case of FC treatment, γ-cyhalothrin molecules are evenly dispersed throughout the water with the carrier solvent, facilitating a relatively consistent exposure to the *C. dubia* as it swims through the water column. For the LF and SF, γ-cyhalothrin is mostly associated with the capsules rather than freely suspended, and therefore γ-cyhalothrin exposure to *C. dubia* is probably limited to encounters with the capsules. Assuming a large capsule contains more γ-cyhalothrin than a small capsule, there are fewer LF capsules than SF capsules for a given concentration (treatments were normalized by γ-cyhalothrin content, not capsule count). Therefore, the LF treatment had fewer capsules in suspension than the comparable SF treatment, which would exacerbate the increased response variability associated with capsule-mediated exposures.

The three-dimensional structure of capsules could also play a role in the toxic response. Cladocerans like *C. dubia* use mesh sieves on their appendages to ingest particles ranging from approximately 100 to 5000 nm in diameter as adults, with the preferential uptake occurring near 500 nm [31]. Given that the neonates used in this study are smaller than adults, a preferential uptake of particles under 500 nm is sensible and may result in increased ingestion of the SF when compared to the LF. One study found that nanoencapsulation increased the overall uptake of a pyrethroid in earthworms, but most of the active ingredient remained in the gut rather than being internalized [15]. If this were the case for the *C. dubia*, encapsulation could prevent a toxic response by preventing internalization of γ-cyhalothrin. However, there is evidence that smaller nanoparticles are more readily taken up in the closely related genus Daphnia [32], in which case capsules may increase γ-cyhalothrin internalization if sufficiently small. This notion is supported by the observed increase in toxicity from the SF. If capsule leakage is the mechanism for release, small pesticide capsules would release active ingredients more rapidly than large capsules [33]. Our partitioning experiment did not suggest a difference in γ-cyhalothrin release rate between SF and LF, though enzymatic degradation of capsules in the gut of *C. dubia* could affect the capsule integrity.

Finally, the stability of the capsules in suspension could be influenced by the neonates. While our preparation of the SF and LF samples involves a 48 h settling period before conducting the immobilization assay, the *C. dubia* could exert an effect on the particle stability. Other researchers have found that protein exudates from Daphnia can prompt the agglomeration of nanoparticles within a matter of hours [34]. Agglomeration of the pesticide capsules could cause them to drop out of suspension and sink to the bottom of the water column where they are unlikely to be ingested by filter feeders. Agglomeration state could not be determined here because DLS is not sensitive enough to verify the size of the pesticide capsules at the low particle concentrations used in our toxicity assessments.

## 4. Conclusions

Innovations in pesticide formulation are important for manufacturers, who benefit from the economic opportunity associated with pesticide sales, as well as other stakeholders concerned with environmental impacts caused by pesticide use. The advantages of encapsulation technologies should not be understated, and combining these features with the recent developments in nanoenabled products is becoming a fruitful area of research. As these investigations continue, it would be mutually beneficial for risk assessors and pesticide manufacturers to minimize the barriers of proprietary technology so that studies like this can result in more effective products that reduce environmental impacts. With the litany of nanoscale capsule designs available, identifying and describing their effects on active ingredients is an important pathway towards innovative pesticide products. In future studies, attention to the rates of release and desorption of active ingredients from nanoparticle carriers, and subsequent incorporation into risk assessment frameworks, will be a valuable contribution to predicting the fate of nanoenabled pesticides.

Besides helping to bolster the pesticide market, keeping pace with the evolution of encapsulated pesticides is vital for regulatory agencies. In this case, we demonstrated that encapsulated formulations may complicate the use of hydrophobicity metrics in modeling endeavors due to time-dependent and size-dependent changes to the active ingredient fate and toxicity. These modifications can alter the risk profile of pesticide products by disrupting foundational assumptions about chemical behavior, such as the reliance upon a K_OW_ value in active ingredient transport. Given concerns surrounding surface water pesticide contamination, recognizing these nuances may lead to more accurate environmental modeling and highlight scenarios in which other ingredients deserve unique attention. 

## Figures and Tables

**Table 1:** Average hydrodynamic diameter (HDD), zeta potential, and polydispersity index (PDI) for the UF, LF, and SF.

Fraction	HDD (nm)	Zeta Potential	PDI
UF	662 ± 9 ^a^	−13.5 ± 2.6	0.291 ± 0.006 ^a^
LF	758 ± 8 ^b^	−11.0 ± 0.8	0.249 ± 0.013 ^b^
SF	449 ± 2 ^c^	−14.6 ± 1.6	0.264 ± 0.004

^a, b, c^ Letters refer to statistical comparison among capsule fractions (Holm–Sidack multiple comparison); *n* = 3; ± standard error.
